# Representation of motion onset and offset in an augmented Barlow-Levick model of motion detection

**DOI:** 10.1007/s10827-012-0393-9

**Published:** 2012-04-13

**Authors:** Timothy Barnes, Ennio Mingolla

**Affiliations:** 1 Program in Cognitive and Neural Systems, Boston University, Boston, MA 02215 USA; 2 Department of Psychology and Center for Computational Neuroscience and Neural Technology (CompNet), Boston University, Boston, MA 02215 USA

**Keywords:** Acceleration, Accretion and deletion, Occlusion, Visual cortex, Visual motion

## Abstract

Kinetic occlusion produces discontinuities in the optic flow field, whose perception requires the detection of an unexpected onset or offset of otherwise predictably moving or stationary contrast patches. Many cells in primate visual cortex are directionally selective for moving contrasts, and recent reports suggest that this selectivity arises through the inhibition of contrast signals moving in the cells’ null direction, as in the rabbit retina. This nulling inhibition circuit (Barlow-Levick) is here extended to also detect motion onsets and offsets. The selectivity of extended circuit units, measured as a peak evidence accumulation response to motion onset/offset compared to the peak response to constant motion, is analyzed as a function of stimulus speed. Model onset cells are quiet during constant motion, but model offset cells activate during constant motion at slow speeds. Consequently, model offset cell speed tuning is biased towards higher speeds than onset cell tuning, similarly to the speed tuning of cells in the middle temporal area when exposed to speed ramps. Given a population of neurons with different preferred speeds, this asymmetry addresses a behavioral paradox—why human subjects in a simple reaction time task respond more slowly to motion offsets than onsets for low speeds, even though monkey neuron firing rates react more quickly to the offset of a preferred stimulus than to its onset.

## Introduction

The human visual system operates in depth, separating even the simplest images into a figure and its background (Rubin 1921). Kinetic occlusion (Michotte et al. 1991; Kaplan 1969) is one figure-ground segregation cue that may be processed early in the visual hierarchy. The ubiquity and primacy of motion processing across species provides some evidence for a low-level kinetic occlusion mechanism: for example, both humans (van Doorn and Koenderink 1982) and bees (Srinivasan et al. 1990) can find edges defined by motion parallax alone; conversely, prey are better hidden when both camouflaged and still (Heatwole 1968). However, the neural mechanisms that process local motion signals, while modeled in animals like the fly (Hassenstein and Reichardt 1956) and rabbit (Barlow and Levick 1965), are not fully understood in primates.

Kinetic occlusion produces discontinuities in the optic flow field, whose saliency increases with surface texture density. A patch of contrast on a far surface will move through the optic flow field until it suddenly stops at the occluding boundary and disappears from view (texture deletion; Kaplan 1969). If the far surface is instead being uncovered, patches of contrast suddenly appear at the occluding boundary (texture accretion). The sudden onset and offset of contrast affects motion discrimination in a way that suggests it produces a strong transient signal (Churan et al. 2009). Accretion and deletion are not necessary for depth ordering from kinetic occlusion (Yonas et al. 1987), but they are *local* cues that have proven useful in computer vision models of depth ordering (Black and Fleet 2000; Feldman and Weinshall 2008). During kinetic occlusion the change in texture is accompanied by the onset and offset of local motion signals. Detection of these motion onsets and offsets may thus be an important early step in kinetic occlusion perception.

Our understanding of motion onset and offset neural mechanisms is guided by reaction time (RT) studies, which have yielded two general results: subjects report their perception of onset and offset after a time that is inversely proportional to the speed of an object while it moves (Dzhafarov et al. 1993; Kawakami et al. 2002), and they respond slightly more slowly to motion offsets than onsets (Kreegipuu and Allik 2007). Monkey neurophysiology studies have searched for sustained acceleration and deceleration signals in visual areas, of which onsets and offsets are extremes. These studies have not found cells in visual motion areas whose tonic firing varies linearly with acceleration, but many cells produce transient responses to both the onset and offset of motion (Lisberger and Movshon 1999). Generalizing to all accelerations, the studies suggest that adaptation of middle temporal area (MT) cell activities to moving stimuli may allow for a population-level representation of acceleration (Priebe and Lisberger 2002; Price et al. 2005; Schlack et al. 2007). This adaptation may also explain the reaction time results mentioned above (Dzhafarov et al. 1993).

We have synthesized these results into a circuit model that detects the unexpected onset or offset of stimulus motion. Based on evidence that Meynert cells in layer six of primary visual cortex (V1) use a nulling inhibition mechanism for motion detection (Livingstone 1998), we use the Barlow-Levick detector (Barlow and Levick 1965) as an elementary motion detector rather than a correlative (Hassenstein and Reichardt 1956) or energy (Adelson and Bergen 1985) model. The output of the Barlow-Levick model is the input to a similar circuit, which prefers strong accelerations/decelerations by inhibiting responses to constant motion, just as the original circuit inhibits against the null direction of motion. The cells in this new model layer respond selectively to stimulus motion onset and offset over a limited range of speeds, the distribution reflecting responses of MT cells to accelerations and decelerations (Schlack et al. 2007). We show that, given a simple model of reaction time (Ratcliff 1978), the speed-dependent response of onset and offset cells also qualitatively explains the difference in human subject reaction times when responding to the onset and offset of stimulus motion (Kreegipuu and Allik 2007). The key model insight is that, in order to produce a positive offset response to an absence of neural activity corresponding to motion, the system must produce excitatory activity (tonic excitation, predictive priming, etc.) that underlies both a faster neural response and a slower behavioral response relative to motion onsets.

## Model specification

**Fig. 1:**
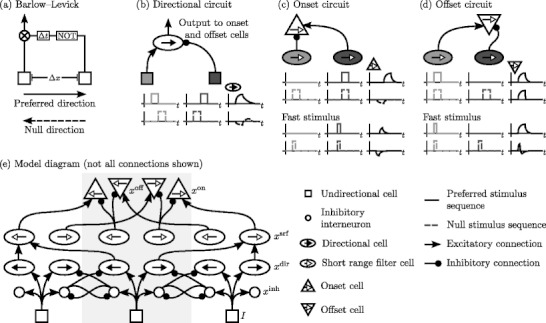
(**a**) Diagram of the Barlow-Levick motion detector, which uses spatially and temporally shifted inhibition to produce directional selectivity. (**b**) An alternative circuit diagram is shown with six idealized plots that demonstrate how the directional cell reacts to different stimulus sequences. The *top row* (*solid lines*) shows that if the left input (*light gray*) is activated before the right input (*dark gray*), the directional cell (*black curve*) responds. The null stimulus sequence (*bottom row*), where an input activates the right cell before the left, produces a negligible cell response. (**c**) An onset cell receives filtered input from the output of (**b**). Its preferred stimulus is a directional input that did not appear previously (onset; top row of plots), while its null stimulus sequence is a directional input that appears behind its position before activating its own position (*second row*). This circuit acts similarly at high speeds, although the cell may not receive input for long enough to become active (*third and fourth rows*). (**d**) An offset cell prefers a directional input that stops short of its own position (offset; top row of plots). Offset cells respond to the null stimulus sequence as well (constant motion; *second row*), but this response diminishes with increasing stimulus speed (*third and fourth rows*). This activity has implications regarding reaction times to motion onsets vs. offsets (Section 3.1). (**e**) A complete model unit at one position, marked by a light gray background and aligned over a central undirectional cell (square symbol at middle of bottom layer), is shown with adjacent directional circuits. The circuit is a combination of motion direction, onset, and offset detectors (**b**)–(**d**). Only those connections are shown that illustrate a scheme to be repeated across a cell type for each position and direction. A short range filter (SRF) between the directional and onset/offset layers silences isolated motion signals in spurious directions that are produced when a stimulus first appears. In order to match known neuroanatomy, inhibitory interneurons are added as a source of model inhibition (Livingstone 1998). The reciprocal inhibition between interneuron pairs may result in more complex model dynamics for simulations with varying contrast strength. These issues are investigated in other studies (Grossberg and Raizada 2000; Raizada and Grossberg 2001)

The model presented here is built on the Barlow-Levick model of directional selectivity (Barlow and Levick 1965), which estimates the spatiotemporal directional derivative of a contrast signal by sampling at two regions separated by time and visual space. The two regions designate the model as a bilocal detector, as opposed to local gradient models or global Fourier mechanisms (van Doorn et al. 1984). In contrast to a bilocal correlation detector (Hassenstein and Reichardt 1956), which produces a signal *only* when both regions contain input activity, the Barlow-Levick detector responds when the later region is excited *unless* the delayed region is excited first (nulling inhibition). A threshold-linear signal function forces the output to respond positively for contrast motion in one direction without responding negatively to the opposite direction, but a threshold-quadratic nonlinearity may be more physiologically accurate (Grzywacz et al. 1990). In our work we reuse the idea of nulling inhibition present in the Barlow-Levick circuit (Fig. 1(a) and (b)) for a circuit detecting motion onset and offset (Fig. 1(c) and (d)). While onset cells are preemptively inhibited by an approaching stimulus, similarly to the Barlow-Levick mechanism, offset cells must instead be excited by an approaching stimulus in order to produce a signal in the absence of a stimulus; inhibition silences this activity when the stimulus continues moving. This reversed temporal ordering is what generates neural and behavioral properties that differ from those of onset cells.

We implement the Barlow-Levick model as a rate-based continuous dynamical system without temporal delays, with cells that occupy discrete positions and interact with spatial nearest neighbors; this simplicity allows for mathematical clarity at the expense of some biological realism, which could be added through spiking dynamics, conductance delays, or more finely graded receptive fields. The model comprises a repeating set of basic spatial units, each unit consisting of eleven cells whose receptive fields are centered on one location *i* (Fig. 1(e)). Cells are also tuned to a single direction *d* (*d* = *l* is leftward motion, *d* = *r* is rightward motion). The internal activity of each cell, $x^{\rm type}_{i,d}$, has a rectified value $[x^{\mathrm{type}}_{i,d}]^+ = \max(x^{\mathrm{type}}_{i,d},0)$ that represents the cell’s firing rate, bounded by the parameter *α*; negative activity corresponds to hyperpolarization. Except for undirectional cells, whose activity time course is explicitly defined as simulation input (in Section 3), the activities of all model cells evolve according to an ordinary differential equation of the following general form:

1 $$ \begin{array}{rll} \tau \dot{x}_{i,d}^{\mathrm{type}} &=& A (0 - x_{i,d}^{\mathrm{type}})\\ &&+ \left(\alpha - x_{i,d}^{\mathrm{type}}\right)(I_{\mathrm{exc}})\\ &&-B \left(\omega + x_{i,d}^{\mathrm{type}}\right)(I_{\mathrm{inh}}). \end{array} $$

The symbols *I*_exc_ and *I*_inh_ in Eq. (1) are replaced in a cell layer-specific manner with expressions for excitatory and inhibitory synaptic influences, respectively; Eq. (2) gives an example. *τ* is a time-scaling constant that controls the rate of system evolution. In the absence of excitatory or inhibitory synaptic input, a cell will passively decay to 0 at rate *A*/*τ*. The second term controls cell excitation, which is bounded by *α*, the maximum allowed activity level of a cell according to the shunting equation (Grossberg 1973). Excitatory inputs from other cells (expression replacing *I*_exc_) are scaled by activity relative to *α*. The inhibitory term contains a gain parameter *B*, a shunting limit term $(\omega + x^{\mathrm{type}}_{i,d})$ for the maximum allowed hyperpolarization level (− *ω*, *ω* ≥ 0), and layer-specific synaptic input functions (expression replacing *I*_inh_) described below. The parameters used in all simulations are given in Table 1. *A* < 1 so that inputs effect a substantial firing rate increase, and *B* > 1 so that the circuit selects only one direction for most inputs. All parameters are in arbitrary units: time can be rescaled by changing *τ*, and activity can be rescaled by multiplying the activity bounds *α* and *ω* by a common factor.

**Table 1:** Model parameters used in Eqs. (2)–(6) for all simulations

Parameter	Value
A	0.1
B	10
*τ*	1
*α*	1
*ω*	0.3

### Barlow-Levick circuit

For simplicity, the input to the model (squares in Fig. 1(e)) is specified as an undirectional contrast signal *I*_*i*_ at positions denoted by the spatial index *i*. The input is explicity defined as a function of time *t* and position *i*, rather than as a differential equation. *Undirectional cells* correspond to cells in magnocellular lateral geniculate nucleus (LGNm), which respond to sudden increases or decreases in contrast (Benardete and Kaplan 1999).

Undirectional cells activate both directional cells and their associated inhibitory interneurons. The interneurons provide nulling inhibition to adjacent positions, where an input would otherwise activate both directions at that position. These interneurons do not use an explicit time delay but instead have a slow passive decay rate, which leaves an activity trace (“delay”) after an input disappears. In some models (Grossberg et al. 2001; Berzhanskaya et al. 2007) directional cells decay quickly, but for simplicity we use the same decay parameter for all model cells.

The activities of *inhibitory interneurons* (small circles in Fig. 1(e)) are modeled by the equation

2 $$ \begin{array}{rll} \tau \dot{x}^{\mathrm{inh}}_{i,d} &=& A \left(0 - x^{\mathrm{inh}}_{i,d}\right)\\ && + \left(\alpha - x^{\mathrm{inh}}_{i,d}\right)I_i\\ && - B\left(\omega + x^{\mathrm{inh}}_{i,d}\right)\left[ x^{\mathrm{inh}}_{j,D} \right]^+\,\,. \end{array} $$

An interneuron spontaneously decays at rate *A*/*τ*. The excitatory term, with shunting limit term *α*, is activated by a connection from the undirectional cell input layer *I*_*i*_. The cell will become active unless inhibited, with gain *B* and lower bound − *ω*, by a signal from an interneuron $x^{\mathrm{inh}}_{j,D}$ in an adjacent position *j* and tuned to the opposite direction *D*. In our one-dimensional implementation, where *i* = 1 is the leftmost cell, for a leftward cell *d* = *l*, *j* = *i* − 1, and *D* = *r*; for a rightward cell, *d* = *r*, *j* = *i* + 1, and *D* = *l*. Interneurons $x^{\mathrm{inh}}_{i,d}$ and $x^{\mathrm{inh}}_{j,D}$ mutually inhibit each other (Fig. 1(e)).

Model *directional cells* (ellipses with filled arrow heads, Fig. 1(e)) correspond to directionally selective Meynert cells in layer six of V1 (Livingstone 1998). The activities of these directional cells are governed by a similar equation to that of inhibitory interneurons:

3 $$ \begin{array}{rll} \tau \dot{x}^{\mathrm{dir}}_{i,d} &=& A \left(0 - x^{\mathrm{dir}}_{i,d}\right)\\ && + \left(\alpha - x^{\mathrm{dir}}_{i,d}\right)I_i\\ && - B\left(\omega + x^{\mathrm{dir}}_{i,d}\right)\left[ x^{\mathrm{inh}}_{j,D} \right]^+\,\,. \end{array} $$

The cell spontaneously decays with rate *A*/*τ* in the absence of other input. It is excited by undirectional inputs *I*_*i*_ towards the upper bound *α* and is inhibited by rectified interneuron inputs $x^{\mathrm{inh}}_{j,D}$ with gain *B* towards the lower activity bound − *ω*. A directional cell $x^{\mathrm{dir}}_{i,d}$ receives the same synaptic inputs as its associated interneuron $x^{\mathrm{inh}}_{i,d}$. The cell is excited by an undirectional input *I*_*i*_ unless inhibited by an interneuron $x^{\mathrm{inh}}_{j,D}$ displaced forward in the directional cell’s preferred direction and tuned to the opposite direction ($x^{\mathrm{dir}}_{i,r}$ is inhibited by $x^{\mathrm{inh}}_{i+1,l}$, $x^{\mathrm{dir}}_{i,l}$ is inhibited by $x^{\mathrm{inh}}_{i-1,r}$). This inhibition prevents a rightward moving stimulus from producing any leftward-tuned directional cell activity, and vice versa.

The filtered output of directional cells becomes the input to model onset and offset cells (described below), just as it forms the basis of some more elaborate motion processing models (Chey et al. 1998; Grossberg et al. 2001; Berzhanskaya et al. 2007).

### Short range filter

Model *short range filter (SRF) cells* (ellipses with empty arrow heads, Fig. 1(e)) correspond to directionally selective cells likely to be found in V1. They have been separately hypothesized as the basis for speed selectivity (Chey et al. 1998) and as MT input subunits that explain motion transparency psychophysics (Qian et al. 1994, Fig. 8), which makes layer four of V1 a likely physiological correlate. The activities of short range filter cells are governed by an equation with only excitatory input (*I*_inh_ = 0):

4 $$ \begin{array}{rll} \tau \dot{x}^{\mathrm{srf}}_{i,d} &=& A \left(0 - x^{\mathrm{srf}}_{i,d}\right)\\ && + \left(\alpha - x^{\mathrm{srf}}_{i,d}\right) 10 \cdot \left[ x^{\mathrm{dir}}_{i,d}\right]^+ \cdot \left[x^{\mathrm{dir}}_{k,d}\right]^+\,\,. \end{array} $$

The cell spontaneously decays with rate *A*/*τ* in the absence of other input. It is excited by the simultaneous presence of directional cell input at its own position $x^{\mathrm{dir}}_{i,d}$ and of directional cell input at a position behind it according to its directional selectivity ($x^{\mathrm{srf}}_{i,r}$ is gated by $x^{\mathrm{dir}}_{i-1,r}$, $x^{\mathrm{dir}}_{i,l}$ is gated by $x^{\mathrm{inh}}_{i+1,l}$). By filtering isolated motion signals and responding only to multiple directional cell activities, the SRF discriminates between constant motion and motion onset/offset, producing a signal that can easily be transformed into an onset/offset signal. The model proposed in this paper, however, never directly compares directional cell and short range filter activities to invert the constant motion signal into an onset/offset signal. When a patch of contrast first appears, directional cells are activated in every direction; the SRF keeps isolated directional cell activities from activating onset/offset cells in directions other than the true stimulus direction of motion. The exact form of the equation is not essential (see Chey et al. 1998, Eqs. (A3)–(A4)) as long as a nonlinearity suppresses small motion signals.

### Onset and offset detectors

Onset and offset model cells act similarly to the Barlow-Levick model in that they signal a large acceleration or deceleration, respectively, unless a nearby spatially and temporally displaced directional signal also occurs. When directional inputs activate neighboring positions in order, the excitation and inhibition to these cells are balanced; otherwise one or the other will become active according to whether the motion has suddenly stopped or started. In the course of constant motion, onset (offset) cells will be preemptively inhibited (excited) before the second input appears. While these model cells are used as comparators for MT speed tuning characteristics (Schlack et al. 2007) in the present work, model units with this connectivity pattern might instead be identified with cells in the second primate visual area (V2), some of which respond selectively to kinetic contours (Marcar et al. 2000).

The firing activity of an *onset cell* (upward-pointing triangle in Fig. 1(e)) at position *i* and tuned to direction *d* evolves according to the equation

5 $$ \begin{array}{rll} \tau \dot{x}^{\mathrm{on}}_{i,d} &=& A \left(0 - x^{\mathrm{on}}_{i,d}\right)\\ && + \left(\alpha - x^{\mathrm{on}}_{i,d}\right)\left[x^{\mathrm{srf}}_{j,d}\right]^+\\ && - B\left(\omega + x^{\mathrm{on}}_{i,d}\right)\left[ x^{\mathrm{srf}}_{i,D} \right]^+\,\,. \end{array} $$

This cell spontaneously decays with rate *A*/*τ*, is excited by directional cell activity directly ahead of the current position $x^{\mathrm{srf}}_{j,d}$ (in our implementation, *j* = *i* + 1 when *d* = *r* and *j* = *i* − 1 when *d* = *l*) towards the upper activity bound *α*, and is inhibited by directional cell activity at its own position $x^{\mathrm{srf}}_{i,d}$ with gain *B* toward the lower activity bound − *ω*. The cell is excited by a nearby SRF signal $x^{\mathrm{srf}}_{j,d}$ unless that signal has already propagated through the onset cell’s own position *i*.

An *offset cell* (downward-pointing triangle in Fig. 1(e)) receives inputs from the same layers as the onset cell but with a complementary pattern of excitation and inhibition, shown in the activity equation

6 $$ \begin{array}{rll} \tau \dot{x}^{\mathrm{off}}_{i,d} &=& A \left(0 - x^{\mathrm{off}}_{i,d}\right)\\ && + \left(\alpha - x^{\mathrm{off}}_{i,d}\right)\left[x^{\mathrm{srf}}_{k,d}\right]^+\\ && - B\left(\omega + x^{\mathrm{off}}_{i,d}\right)\left[ x^{\mathrm{srf}}_{i,d} \right]^+\,\,. \end{array} $$

This cell decays with rate *A*/*τ*, is excited by displaced SRF signals $x^{\mathrm{srf}}_{k,d}$ towards the upper activity bound *α*, and is inhibited by interneurons at its own position $x^{\mathrm{srf}}_{i,d}$ with gain *B* toward the lower activity bound − *ω*. The offset cell will become active if the SRF cell $x^{\mathrm{srf}}_{k,d}$ behind it (*k* = *i* − 1 when *d* = *r* and *k* = *i* + 1 when *d* = *l*) is activated, unless the input continues into its own position *i*.

Activity variable names and connectivities for all of the above cell types are shown in Fig. 1(e).

## Simulations

**Fig. 2:**
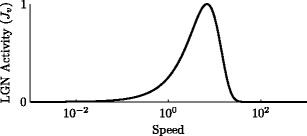
LGN activity *J*_*v*_ as a function of stimulus speed, defined by Eqs. (8)–(10) (Benardete and Kaplan 1999)

The circuit described above should explicitly signal the direction of a motion, its onset, and its offset. The simulations presented below demonstrate the circuit’s responses to undirectional inputs that suddenly start moving at a constant speed before suddenly stopping. All simulations were run on a network with seven positions/model units, making a 77-cell implementation. Each simulation depicts a patch of contrast *K*_*i*,*v*_(*t*) at the second position from the left (*i* = 2) that suddenly begins moving to the right at a constant speed *v* before abruptly stopping at position *i* = 6:

7 $$ K_{i,v}(t) = \begin{cases} \delta \big[ \lfloor t \cdot v \rfloor - (i - 2) \big] & \mbox{for} \; 2 \le i \le 6 \\ 0 & \mbox{for} \; i \in \{1, 7\} \end{cases} \; , $$

where *δ* is the Kronecker delta and $\lfloor\cdot\rfloor$ is the floor function. This simulated contrast patch activates undirectional cells by an amount *J*_*v*_ (Fig. 2), programmed as the response of LGNm cells to a stationary Gabor luminance patch with spatial frequency *f*_*S*_ and contrast modulated at temporal frequency *ω* (Benardete and Kaplan 1999, modulus of Eqs. (3) and (4)):

8 $$ J_v = F \sqrt{\left(1-\frac{2 H_S - H_S^2} {1 + \left(\omega\tau_S\right)^2}\right) \left(1 + \left(\omega\tau_L\right)^2\right)^{-N_L}} \; ; $$

9 $$ \tau_S = \frac{T_0}{1 + \left(c / C_{1/2}\right)^2} \; ; $$

10 $$ \omega = 2\pi \cdot f_S \cdot v \; , $$

where *F* = 2.206 was chosen for maximum activity $J_v^{\mathrm{max}} = 1$, *H*_*S*_ = 1.00, $\tau_L = 1.68\cdot10^{-3}$ s, *N*_*L*_ = 25.50, $T_0 = 4.496\cdot10^{-3}$ s, and *C*_1/2_ = 0.048 (Benardete and Kaplan 1999, Table 2, Median/ALL). $f_S = 2.181\;\circ^{-1}$ as an example optimal spatial frequency (Benardete and Kaplan 1999, Fig. 7) and *c* = 0.1. By using the response to a contrast patch that is modulated only in the temporal domain, we are assuming that LGN responds similarly both to temporally modulating and to phase-rolling Gabor patches. This should be approximately true if LGN receptive fields are space-time separable. We also assume that the contrast configuration used as a stimulus is not distorted as it shifts, which requires uniform lighting and a smooth surface without specular reflections.

The undirectional model input *I*_*i*_, for a given speed *v*, is a combination of the contrast patch position *K*_*i*,*v*_(*t*) and undirectional cell activity *J*_*v*_:

11 $$ I_i(t) = J_v \cdot K_{i,v}(t) \; . $$

**Fig. 3:**
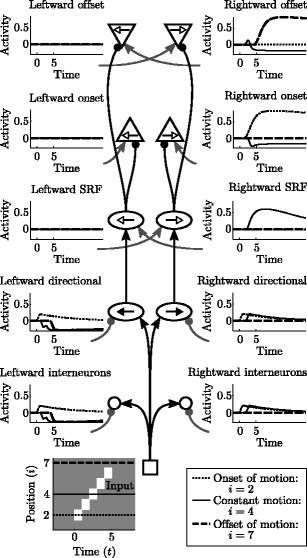
A simulation example. The *bottom* space/time plot shows how undirectional cells (represented by a square in the central circuit diagram) are activated as a bar of contrast moves to the right (increasing position index *i*). At the onset of motion (*dotted lines* in all graphs, *t* = 0, *i* = 2) left and right directional cells begin to activate, as well as the rightward onset cell. Leftward motion signals are also produced but are gated by the SRF, keeping them from producing leftward onsets or offsets. During constant motion (*solid lines*, only activity at position *i* = 4 shown) the input is active at each position for 1 unit of simulation time. Only rightward motion is signaled; leftward directional cells and interneurons are inhibited, so they can neither signal motion nor inhibit rightward motion signals. A small rightward offset response is also produced. After motion offset (*dashed lines*, *t* = 5, *i* = 7) only a rightward offset cell becomes active

An example simulation is shown in Fig. 3. All simulations were numerically integrated by MATLAB’s ode45 function (MATLAB 2010), which implements an adaptive time-step 4th-order Runge Kutta solver.

The sudden onset of a stimulus produces an onset signal as well as directional signals in both directions away from the onset position (Fig. 3, dotted lines). Once the input activity continues to the right, however, the signal becomes directional because leftward directional cells are preemptively inhibited before the input arrives (Fig. 3, solid lines). Offset cells respond vigorously when the stimulus ends (Fig. 3, dashed lines), but they also give a response during constant motion. This small, “incorrect” signal reduces the reliability of offset signals. The size of both correct and spurious (incorrect) responses vary as a function of stimulus speed—a relationship investigated in Section 3.1.

### Speed-dependent model responses

The Barlow-Levick detector is sensitive to speed, just as a motion energy filter responds to stimuli within a limited speed range (Simoncelli and Heeger 1998). Onset and offset cell activities also change in amplitude with a change in stimulus speed: at speeds for which directional cells vigorously respond, onset/offset cells are also more active, both when the stimulus begins/ends (correct) and when the stimulus moves uniformly (incorrect). We measured this speed dependence with a selectivity measure that increases with correct activity and decreases with incorrect activity. In order to do this, we simulated the lumped activities of three distinct theoretical neuron populations that have different input connectivities for accumulating evidence of motion onset, a particular motion direction, and motion offset. We assume that evidence accumulating populations exist or are dynamically constructed for different tasks; we implement only those that are sensitive to aspects of the stimuli used in the presented simulations. Each accumulator population is excited by “correct” activity and inhibited by “incorrect” cell activity. *Selectivity* is defined as the maximum activity this evidence accumulator population reaches, which roughly corresponds to the time it takes to reach a threshold activity after taking into account unmodeled brain processes such as competition between evidence accumulators and higher-level motion grouping processes. Onset and offset selectivities are inverted in Section 3.2 to create a measure of the model’s reaction time to the presence of that stimulus aspect. This measure captures our assumption that some other brain region which controls the motor response of a subject in a reaction time experiment accumulates evidence for when a moving stimulus has changed while habituating to “incorrect” cell activity (Dzhafarov et al. 1993).

The evidence accumulator for simulation stimulus motion onset is excited when the rightward onset cell at position *i* = 2 is activated ($x^{\mathrm{on}}_{2,r}$) and is inhibited when onset activity occurs at any other position ($\sum_{i\neq 2}x^{\mathrm{on}}_{i,r}$). The activity level of this accumulator population is modeled by an ordinary differential equation similar to Eq. (1), differing only in the parameter *C*, which allows evidence to be accumulated over longer time periods:

12 $$ \begin{array}{rll} C \cdot \tau \dot{y}^{\mathrm{on}} &=& A(0 - y^{\mathrm{on}})\\ &&+\left(\alpha - y^{\mathrm{on}} \right)\left[x^{\mathrm{on}}_{2,r}\right]^+\\ &&- B(\omega + y^{\mathrm{on}})\sum\limits_{i\neq2} \left[x^{\mathrm{on}}_{i,r}\right]^+\,\,\,. \end{array} $$

**Fig. 4:**
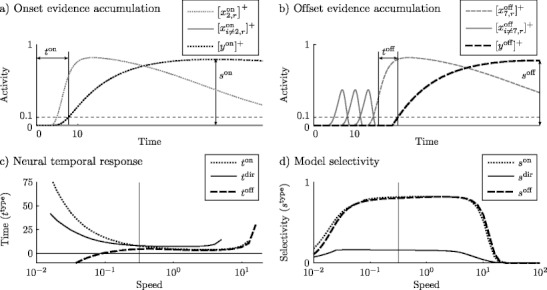
(**a**) Activity traces of the model onset evidence accumulator *y* ^on^ (*dotted black line*) and its excitatory (*dotted gray line*) and inhibitory (*solid gray line*) inputs (Eq. (12)). Because model onset cells are preemptively inhibited, most of them are silent over simulations run at many different speeds; that is, the value of $[x^{\mathrm{on}}_{i\neq 2,r}]^+ = 0$. Neural onset latency *t* ^on^ is defined as the time taken from actual stimulus onset to an evidence accumulator activity level *y* ^on^ = 0.1. Onset selectivity *s* ^on^ is the maximum evidence accumulator activity level reached over the simulation. (**b**) A similar plot showing model offset evidence accumulator *y* ^off^ activity (*dashed black line*) against its excitatory (*dashed gray line*) and inhibitory (*solid gray line*) inputs (Eq. (16)). The neural response time *t* ^off^ is measured relative to the time of stimulus motion offset. (**c**) Neural response latencies for onset (*dotted*), direction (*solid*), and offset (*dashed*) evidence accumulators. The vertical line in the middle of the plot marks the speed for the simulation shown in (**a**) and (**b**). Onset and direction latencies are high at low speeds, but offset latencies decrease to where offset signals predict the actual offset—a consequence of preemptive excitation received by offset cells. Onset and offset latencies continue to diverge at lower speeds, but the graph is cut off for readability. (**d**) Model selectivities for a range of stimulus speeds. The vertical line in the middle of the plot again marks the simulation shown in (**a**) and (**b**). Onset and offset selectivities are high relative to directional selectivity because of the input nonlinearity that gates SRF activity. Onset and offset selectivities are very similar except for a slight lowering of offset selectivity *s* ^off^ at low stimulus speeds

In all simulations, *C* = 10; the values of the other parameters used in this and other evidence accumulator equations are listed in Table 1. A sample simulation of this accumulator is plotted against its inputs in Fig. 4(a). The figure also demonstrates the measurement of a neural response time to motion onset *t* ^on^ as the time from stimulus motion onset to the evidence accumulator activity *y* ^on^ crossing a threshold of 0.1. Model onset selectivity *s* ^on^ is the maximum activity reached by the onset evidence accumulator,

13 $$ s^{\mathrm{on}} = \max_t \left[ y^{\mathrm{on}}(t) \right] \; . $$

Both *s* ^on^, a measure of the model’s perceptual response, and *t* ^on^, a measure of the neural response, are shown for a single speed in Fig. 4(a).

The evidence accumulator population for motion in the rightward direction is excited by a rightward direction cell chosen at a position where constant motion occurs ($x^{\mathrm{dir}}_{6,r}$) and is inhibited by leftward motion at that same position ($x^{\mathrm{dir}}_{6,l}$). Its activity is modeled by the equation

14 $$ \begin{array}{rll} C \cdot \tau \dot{y}^{\mathrm{dir}} &=& A(0 - y^{\mathrm{dir}})\\ &&+\left(\alpha - y^{\mathrm{dir}}\right)\left[x^{\mathrm{dir}}_{6,r}\right]^+\\ &&- B(\omega + y^{\mathrm{dir}}) \left[x^{\mathrm{dir}}_{6,l}\right]^+\,\,\,. \end{array} $$

The maximum activity of *y* ^dir^ is used as a measure of directional selectivity

15 $$ s^{\mathrm{dir}} = \max\limits_t \left[ y^{\mathrm{dir}}(t) \right] \; . $$

The rightward offset cell at position *i* = 7 ($x^{\mathrm{off}}_{7,r}$) excites the evidence accumulator population for motion offset, while offset signals at all other positions ($\sum_{i\neq 7}x^{\mathrm{off}}_{i,r}$) inhibit it. These signals are combined in the following evidence accumulator equation:

16 $$ \begin{array}{rll} C \cdot \tau \dot{y}^{\mathrm{off}} &=& A(0 - y^{\mathrm{off}})\\ &&+\left(\alpha - y^{\mathrm{off}}\right)\left[x^{\mathrm{off}}_{7,r}\right]^+\\ &&- B(\omega + y^{\mathrm{off}})\sum\limits_{i\neq7} \left[x^{\mathrm{off}}_{i,r}\right]^+\,\,\,. \end{array} $$

The maximum activity of the offset evidence accumulator defines the model’s offset selectivity:

17 $$ s^{\mathrm{off}} = \max\limits_t \left[ y^{\mathrm{off}}(t) \right] \; .$$

A neural response time to stimulus motion offset is also measured as the amount of time from stimulus motion offset to the offset accumulator reaching an activity threshold of 0.1; both measures are shown with a sample simulation in Fig. 4(b).

Figure 4(c) plots the temporal delay between a stimulus event and the generation of a response from the corresponding evidence accumulator. The time of the generated response is defined as the time at which the evidence accumulator activity *y* ^type^ is greater than 0.1. This latency generally decreases with increasing speed for the neural response to stimulus onset *t* ^on^ and the establishment of directionality in a local area *t* ^dir^. Because stimulus offset signals are preemptively generated, the latency actually decreases and can occur before actual stimulus motion offset at low speeds. The neural response to stimulus motion offset has a lower latency than the neural response to stimulus motion onset in this model, especially at low speeds, in accordance with the results of a VEP analysis paired with a reaction time experiment (Kreegipuu and Allik 2007). At high speeds all latencies increase because the stimulus moves too quickly to strongly activate the circuit.

Figure 4(d) shows a measure of model selectivities *s* ^type^ for simulations run with different stimulus speeds. Model selectivity corresponds to peak evidence accumulator activity over a simulation (Eqs. (12)–(17)). All selectivities are limited at high and slow stimulus speeds because LGN does not respond to these stimuli (Fig. 2), and at high speeds the input never remains at a position long enough to activate model cells. Onset and offset selectivities are generally higher than directional selectivity because the nonlinear input term in the SRF (Eq. (4)) expands its directional input activities. “Incorrect” offset signals are large at low stimulus speeds (Fig. 4(b), solid gray curves), which decreases the evidence for an actual motion offset and lowers offset selectivity *s* ^off^ relative to onset selectivity *s* ^on^. At high stimulus speeds, the input is less directional because interneurons have no time to strongly inhibit the opposite direction; directional cells thus have low activity except at the last stimulus position, where no directional competition occurs. This asymmetry boosts offset selectivity relative to onset selectivity at high speeds.

**Fig. 5:**
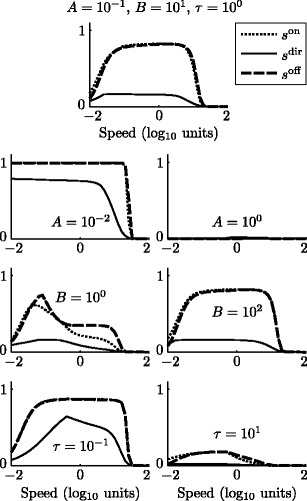
Changes in model selecitivity as a function of parameter choice over two orders of magnitude. The *top row* shows the parameters used for simulations presented in the present article. Changing the relative strength of passive decay (*A*, *second row*) affects the equilibrium value of all cells (height) as well as the ability of the circuit to hold an inhibitory signal long enough to “remember” the stimulus direction; this can expand or contract the speed range over which the circuit operates. Changing the relative strength of inhibition (*B*, *third row*) can reduce or strengthen selectivity; when *B* is small, all directional cells simultaneously become active, which produces stimulus-inappropriate model responses that lower selectivity. Changing the equation time constant (*τ*, *bottom row*) brings the model selectivity characteristics more (small *τ*) or less (large *τ*) in line with the speed-dependent input activation level *J*_*v*_ (Fig. 2)

To disambiguate the effect of circuit connectivity with the stimulus or parameter choices, selectivity curves were calculated for a range of parameters centered around those used in all other simulations. Figure 5 shows how the parameters *A*, *B* and *τ* affect the three selectivity curves described in Eqs. (13), (15) and (17). The relative decay rate parameter *A* controls both the maximum activity of model cells and the length of decaying memory traces; a small *A* produces large peak activities and high selectivity for very slow speeds, while a large *A* silences all model cells. The relative inhibition parameter *B* controls how well a directional stimulus can produce selective responses by silencing inappropriate ones; a small *B* produces high spurious activity and lowered selectivities at most speeds, while a large *B* ensures that these “incorrect” signals are silent across most speeds. The model cell rate parameter *τ* shifts the speed-dependent response characteristcs of model cells; a small *τ* means that cells are more responsive to higher speeds, subject to the input energy envelope established by LGNm (*J*_*v*_, Fig. 2), and a large *τ* means that cells are more responsive to lower speeds, in a range where LGN is unresponsive.

Figure 5 shows that the speeds for which offset selectivity is greater than, approximately equal to, or lower than onset selectivity vary widely over the parameter space. For the parameters tested, however, onset selectivity is never higher than offset selectivity at low speeds, and onset selectivity is never lower than offset selectivity at high speeds, a trend that may be generically true by the model’s architecture. We have chosen a set of parameters that allows for a slight separation between onset and offset selectivities at low speeds and has high selectivity over a wide range of speeds.

### Reaction times

Behavioral data on the perception of motion onset and offset comprises a set of reaction time studies that find an inverse relationship between stimulus speed and response time: the faster the stimulus moves, the shorter the response time of subjects recognizing that the stimulus changed (Dzhafarov et al. 1993; Kawakami et al. 2002; Kreegipuu and Allik 2007). These relationships take the general form *RT* = *c* ·*v* ^− *β*^ + *r*, where *v* is the velocity of the stimulus when it moves, *β* is a parameter controlling the convergence of reaction time to its minimum (generally chosen between 0.5 and 1), *c* is a scaling parameter, and *r* is an additive parameter independent of velocity.

The speed tuning of model onset/offset cell responses can be used to make a measure of model reaction time, which we take to be inversely related to model selectivity. According to a diffusion model of decision making (Ratcliff 1978), mean reaction time is inversely related to the rate of evidence accumulation in cases of low noise. We approximate this accumulation rate by measuring the peak activity of a model evidence accumulator defined in Section 3.1 (Eqs. (12)–(17)). Model reaction times are estimated as the inverse of the onset and offset selectivities *s* ^on^ and *s* ^off^,

18 $$ \mathrm{RT}^{\mathrm{type}}(v) = \frac{c}{s^{\mathrm{type}}{}(v/w)} + r \; , $$

where the inverse is converted from model speed to physical units (*w* = 10°/s), scaled to convert from selectivity to a perceptual decision reaction time (*c* = 100 ms), and vertically shifted to account for a speed-independent motor response (*r* = 175 ms).

**Fig. 6:**
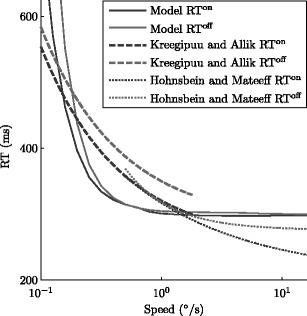
An estimate of model reaction times as a function of stimulus speed, given as the inverse of model onset and offset selectivity (Eq. (18)). This estimate is compared to fits of human psychophysical data, one result fit with the same exponent *β* for onset and offset (Kreegipuu and Allik 2007), and one result fit with different exponents (Hohnsbein and Mateeff 1992). The model qualitatively fits the data in that it decreases with increasing stimulus speed and is biased towards slower responses to stimulus motion offsets than to motion onsets

Figure 6 plots model reaction time against two independent measurements for simple reaction time to a moving random dot field that suddenly starts or stops moving, one fit with the same exponent for onset and offset (Kreegipuu and Allik 2007) and one fit with a separate exponent for each (Hohnsbein and Mateeff 1992). The model reaction time captures the qualitative trends of the data: reaction time decreases with increased speed, and reaction time to stimulus motion offset is slightly delayed relative to that of stimulus motion onset. This is in contrast to neural response time, where cells that register motion offsets respond before those that register motion onsets (Fig. 4(c)). The quantitative fit is poor, however, which means that the gradual curvature of measured reaction time curves is likely produced by a mechanism absent from our model. The simulated circuit will also respond slowly to changes in fast-moving stimuli, contrary to human reaction time data (Kawakami et al. 2002), a discrepancy we address in Section 4.3.

## Discussion

We have presented an analysis of an augmented Barlow-Levick detector and its speed-dependent selectivity to stimulus direction, onset, and offset. The Barlow-Levick detector uses spatially offset inhibition and temporally delayed excitation to select against contrast moving in the circuit’s null direction. Model onset detectors repeat this mechanism, but model offset detectors require temporally delayed inhibition, which produces a preemptive excitation that both generates a positive response to a lack of stimulus and a false offset signal during constant motion, especially at low speeds. Directional, onset and offset cells can all be interpreted as taking successive spatiotemporal directional derivatives of a position signal through bilocal sampling of position (undirectional cell) or velocity (directional cell) signals. Simulation results show that, for some parameter choices, this extended detector is relatively sensitive to the onset of motion at slower speeds and sensitive to the offset of motion at higher speeds, which is consequently reflected in model reaction time (Table 2). The general shape of the reaction time curve shown in Fig. 6 is a result of the model’s LGN response to temporal frequencies (Fig. 2), while the relative displacement of onset and offset curves results from the preemptive inhibitory or excitatory input to onset and offset cells, respectively. The model predicts that cells exist somewhere relatively early in the cortical motion pathways which give an enhanced response, measured by single-unit recordings, if probed by stimuli such as the following: a rightward moving dot approaches a certain retinal position and stops just outside of the classical receptive field of an otherwise conventional-appearing motion-sensitive unit tuned to leftward motion. Alternatively, the cell might be a hitherto unreported cell type that responds exclusively to the offset of motion within a small visual field area.

**Table 2:** Summary of simulation results

Stimulus quality	Neural latency	RT-slow	RT-fast
Direction	Long	N/A	N/A
Onset	Longer	Long	Short
Offset	Short	Longer	Short

The model presented in this paper is built upon a bilocal motion detector; while we have used the Barlow-Levick model (Barlow and Levick 1965) based on evidence for its existence in primate visual areas (Livingstone 1998), the onset/offset layer will produce qualitatively similar results with any related motion detector, such as a correlative (Hassenstein and Reichardt 1956) or motion-energy (Adelson and Bergen 1985) model. Our motion detection scheme was derived from and can also form the basis of more complicated motion-processing models (Chey et al. 1998). The output of these motion detectors are gated by a short range filter before being used as the input to onset/offset cells. In our model the short range filter keeps spurious motion signals produced during motion onset from activating onset/offset cells, but it has been previously theorized for other reasons such as forming a speed-sensitive basis (Chey et al. 1998) and explaining psychophysical responses to transparent motion (Qian et al. 1994).

Our model of onset and offset cell connectivity is similar to a model of pigeon pretectal nucleus cell activities (Zhang et al. 2005). The Zhang et al. model explains how sustained cell activities can arise that linearly vary with stimulus acceleration rate. Their model input is a directional cell whose activity linearly increases as a result of some accelerating stimulus within the directional cell’s receptive field; this implies that stimulus speed changes appreciably while the stimulus is within the cell’s receptive field. Our model instead describes transient cell dynamics that occur when the stimulus speed changes dramatically across adjacent directional cell receptive fields. These two sets of results suggest that the same connectivity may produce either set of dynamics, contingent on the underlying directional cell properties.

### Neurophysiology

Jumps in stimulus speed are associated with the perception of acceleration; the sudden appearance and constant movement of an object is perceived to decelerate from a faster speed, as if it were shot out of a cannon (Runeson 1974). The neural correlates of acceleration perception, however, have proven more elusive than those for motion itself. MT cell responses, for example, are generally insensitive to the rate of an accelerating stimulus (Price et al. 2005); attempts to explain acceleration tuning have so far focused on the response of a population of MT neurons with different rates of adaptation (Priebe and Lisberger 2002; Price et al. 2005; Schlack et al. 2007). Acceleration-sensitive cells have been found neither in cat V1, V2, nor in the posteromedial lateral suprasylvian area (PMLS) (Price et al. 2006). Neurons with analog sensitivity to acceleration have been found in the pigeon pretectal nucleus (Cao et al. 2004), which has been modeled by a similar mechanism to ours (Zhang et al. 2005) and may correspond well to neurons in the superior colliculus and other areas involved in retinal slip during smooth pursuit eye movements in primates.

The perception of kinetic contours, however, involves the detection of speed jumps, which produce transient responses from retinal cells in the tortoise (Thiel et al. 2007) to MT cells in the primate (Lisberger and Movshon 1999). Recorded transient responses to motion offset have always been decrements in firing rate, while our model predicts that cells exist whose firing rate increases at the offset of motion in a visual location displaced from their classical receptive field. While MT cells are too sensitive to motion to respond to motion discontinuities (Marcar et al. 1995), area V2 is both direction selective (Lu et al. 2010)) and selective for kinetic contours (Marcar et al. 2000). Possible neural analogs of model onset/offset detectors, then, could either be MT cells because they are the primary output of V1 Meynert and stellate cells (Maunsell and van Essen 1983), V2 cells because they receive directional input from V1 and respond to kinetic contours, a subset of cells in V1 layer six that receive both lateral input from layer six cells and feedback from layer four (Callaway 1998), or even cells in the superior colliculus.

### Speed-dependent model responses

Onset and offset cell responses to constantly moving stimuli differ because their connectivity produces different responses to the same null stimulus sequence (Fig. 1(c) and (d)). Because offset cells are preemptively excited during constant stimulus motion, they activate selectively at speeds that are fast enough to only weakly activate directional cells. Depending on chosen model parameters, this biases offset cell speed tuning (Section 3.1) towards higher speeds than that of onset cells. This configuration of speed tunings is similar to MT cell speed tuning when presented with speed ramp stimuli (Schlack et al. 2007). The speed tuning bias of MT cells towards higher speeds for decelerating stimuli and lower speeds for accelerating stimuli is usually attributed to an adaptation effect (Price et al. 2005; Schlack et al. 2007), which may also be a general mechanism for creating an offset or rebound response that increases with stimulus strength (Carpenter and Grossberg 1981; Francis et al. 1994; Baloch et al. 1999). Transient MT cell responses are also tuned to slightly higher speeds than sustained responses, which could produce a shifted peak in their difference towards higher speeds (Lisberger and Movshon 1999, Fig. 3). One challenge for our model, however, is that onset latencies decrease with increased speed (Lisberger and Movshon 1999, Fig. 5); offset latencies are not reported. False model offset cell signaling occurs when the cell receives an excitatory input for a significant amount of time before it is inhibited. If this excitatory input arrives later for slower stimuli, then the excitatory and inhibitory signals can be better matched, which produces less false signaling and correspondingly less bias in offset cell speed tuning.

Because the speed tuning of the proposed model relies on the amount of undirectional input that either saturates or silences the circuit, this speed tuning can be modulated by contrast strength, reflected in undirectional cell activity level. While the perception of speed is dependent on contrast (Thompson 1982), we believe contrast will minimally affect the proposed circuit for most speeds because the neural correlates of undirectional cells (LGNm) exhibit strong contrast gain control (Benardete and Kaplan 1999). A more careful modeling study of speed tuning and speed discrimination, built on the Barlow-Levick circuit, explores this relationship with contrast in more detail (Chey et al. 1998).

### Reaction times

If the neural correlates of model onset/offset cell populations strongly influence perceptual performance on simple reaction time tasks for the onset and offset of motion, then according to one reaction time model (Ratcliff 1978), responses should be inversely related to the “drift rate” of evidence accumulation. This inverse relation assumes that decision-making areas directly accumulate evidence from early visual areas, an idea which has some support (Shadlen and Newsome 2001). We assume that the activity level of evidence accumulators is inversely related to reaction time and that the accumulators compete with each other for access to their preferred decision and motor response. Our simulations qualitatively fit reaction time data (Kreegipuu and Allik 2007), but the fit is improved when using an inverse exponent *β* closer to 0.5 (not shown). This suggests either that the decision making process is noisy, which flattens the reaction time curve, or that reaction time is not dependent on early visual areas, but rather on areas that are selective for more complicated stimuli (Dzhafarov et al. 1993).

The neural response timing to preferred stimulus offset has been shown to be faster and more consistent within and across stimulus variations than the neural response to preferred stimulus onset (Bair et al. 2002). This has recently been found to have psychophysical consequences: subjects can better discriminate between two gratings that stop moving at different times than between two gratings that start moving at different times (Tadin et al. 2010, Experiment 2). On one hand, this discrimination result is nominally unrelated to manual reaction time; the smaller discrimination thresholds in offset timing asynchrony further highlight the paradox that a reliable signal used in a discrimination task also produces slower reaction times (Kreegipuu and Allik 2007). On the other hand, our model predicts that the transformation from an offset in one population to an active transient signal in another cell population is inherently noisy, which might affect both manual reaction times and discrimination tasks. The referenced discrimination task, however, was performed at a speed of high selectivity for the motion system (4.8°/s), where our model predictions apply mainly at slow stimulus speeds.

The proposed model has longer reaction times for motion offset at slow speeds than for high speeds. While this effect was not always reported for reaction time studies over large speed ranges (Dzhafarov et al. 1993; Kawakami et al. 2002), it has been reported for studies of reaction time at the lowest speeds detectable by human subjects (Kreegipuu and Allik 2007). This difference was reported to be primarily a vertical shift, which corresponds to similar speed tunings but different selectivities. This model suggests that onset and offset selectivities are different mainly in speed tuning ranges, which would correspond to a horizontal shift in reaction time curves.

Because directional cells are not activated in this model by extremely fast stimuli, reaction times are expected to increase again at high speeds (uptick in Fig. 6(d)). Subjects perform well on both motion onsets and offsets at high speeds, with a negligible increase in reaction time for dots moving at 500°/s (Kawakami et al. 2002). At these speeds, however, a sudden stimulus change will produce a salient signal in other, undirectional circuits that can drive the perceptual and motor response. For example, a static noise image replaced by snow on a cathode ray tube display is a detectable stimulus change that does not create a consistent directional percept. This discrepancy may also be accounted for by using a more sophisticated population model, where small subpopulation responses, such as those from cells that respond selectively to high speeds (> 100°/s), are enhanced relative to the rest of the population.

The detection of motion onset or offset, while possibly the basis of kinetic contour perception, is not necessarily its equivalent. Multiple local events have to be spatially integrated to produce a contour defined by local changes like speed jumps (Shipley and Kellman 1994). Even for displays containing motion onsets and offsets, a perception of occlusion and amodal persistence occurs only when speed jumps occur for texture elements on *only one* side of a boundary (Kaplan 1969). A better understanding of the ecological constraints of kinetic occlusion will help guide research on the mechanisms that link neural responses to motion onsets and offsets to the amodal perception of kinetically occluded surfaces.

## References

[CR1] Adelson EH, Bergen JR (1985). Spatiotemporal energy models for the perception of motion. Journal of the Optical Society of America A.

[CR2] Bair W, Cavanaugh JR, Smith MA, Movshon JA (2002). The timing of response onset and offset in macaque visual neurons. The Journal of Neuroscience.

[CR3] Baloch AA, Grossberg S, Mingolla E, Nogueira CAM (1999). Neural model of first-order and second-order motion perception and magnocellular dynamics. Journal of the Optical Society of America A.

[CR4] Barlow HB, Levick WR (1965). The mechanism of directionally selective units in rabbit’s retina. The Journal of Physiology.

[CR5] Benardete EA, Kaplan E (1999). The dynamics of primate M retinal ganglion cells. Visual Neuroscience.

[CR6] Berzhanskaya J, Grossberg S, Mingolla E (2007). Laminar cortical dynamics of visual form and motion interactions during coherent object motion perception. Spatial Vision.

[CR7] Black MJ, Fleet DJ (2000). Probabilistic detection and tracking of motion boundaries. International Journal of Computer Vision.

[CR8] Callaway EM (1998). Local circuits in primary visual cortex of the macaque monkey. Annual Review of Neuroscience.

[CR9] Cao P, Gu Y, Wang S-R (2004). Visual neurons in the pigeon brain encode the acceleration of stimulus motion. The Journal of Neuroscience.

[CR10] Carpenter GA, Grossberg S (1981). Adaptation and transmitter gating in vertebrate photoreceptors. Journal of Theoretical Neurobiology.

[CR11] Chey J, Grossberg S, Mingolla E (1998). Neural dynamics of motion processing and speed discrimination. Vision Research.

[CR12] Churan J, Richard AG, Pack CC (2009). Interaction of spatial and temporal factors in psychophysical estimates of surround suppression. Journal of Vision.

[CR13] Dzhafarov EN, Sekuler R, Allik J (1993). Detection of changes in speed and direction of motion: Reaction time analysis. Perception & Psychophysics.

[CR14] Feldman D, Weinshall D (2008). Motion segmentation and depth ordering using an occlusion detector. IEEE Transactions on Pattern Analysis and Machine Intelligence.

[CR15] Francis G, Grossberg S, Mingolla E (1994). Cortical dynamics of feature binding and reset: Control of visual persistence. Vision Research.

[CR16] Grossberg S (1973). Contour enhancement, short term memory, and constancies in reverberating neural networks. Studies in Applied Mathematics.

[CR17] Grossberg S, Mingolla E, Viswanathan L (2001). Neural dynamics of motion integration and segmentation within and across apertures. Vision Research.

[CR18] Grossberg S, Raizada RDS (2000). Contrast-sensitive perceptual grouping and object-based attention in the laminar circuits of primary visual cortex. Vision Research.

[CR19] Grzywacz NM, Amthor FR, Mistler LA (1990). Applicability of quadratic and threshold models to motion discrimination in the rabbit retina. Biological Cybernetics.

[CR20] Hassenstein B, Reichardt W (1956). Systemtheoretische analyse der zeitreihenfolgen und vorzeichenauswertung bei der bewegungsperzeption des rüsselkäfers chlorophanus. Zeitschrift für Naturforschung.

[CR21] Heatwole H (1968). Relationship of escape behavior and camouflage in anoline lizards. Copeia.

[CR22] Hohnsbein J, Mateeff S (1992). The relation between the velocity of visual motion and the reaction time to motion onset and offset. Vision Research.

[CR23] Kaplan GA (1969). Kinetic disruption of optical texture: The perception of depth at an edge. Perception & Psychophysics.

[CR24] Kawakami O, Kaneoke Y, Maruyama K, Kakigi R, Okada T, Sadato N (2002). Visual detection of motion speed in humans: Spatiotemporal analysis by fMRI and MEG. Human Brain Mapping.

[CR25] Kreegipuu K, Allik J (2007). Detection of motion onset and offset: Reaction time and visual evoked potential analysis. Psychological Research.

[CR26] Lisberger SG, Movshon JA (1999). Visual motion analysis for pursuit eye movements in area MT of macaque monkeys. The Journal of Neuroscience.

[CR27] Livingstone MS (1998). Mechanisms of direction selectivity in macaque V1. Neuron.

[CR28] Lu HD, Chen G, Tanigawa H, Roe AW (2010). A motion direction map in macaque V2. Neuron.

[CR29] Marcar VL, Raiguel SE, Xiao D, Orban GA (2000). Processing of kinetically defined boundaries in areas V1 and V2 of the macaque monkey. Journal of Neurophysiology.

[CR30] Marcar VL, Xiao D-K, Raiguel SE, Maes H, Orban GA (1995). Processing of kinetically defined boundaries in the cortical motion area MT of the macaque monkey. Journal of Neurophysiology.

[CR31] MATLAB (2010). *Version 7.10 [Computer software]*. Natick, MA: The Mathworks.

[CR32] Maunsell JHR, van Essen DC (1983). The connections of the middle temporal visual area (MT) and their relationship to a cortical hierarchy in the macaque monkey. The Journal of Neuroscience.

[CR33] Michotte A, Thinès G, Crabbé G, Thinès G, Costall A, Butterworth G (1991). Amodal completion of perceptual structures. Michotte’s experimental phenomenology of perception.

[CR34] Price NSC, Crowder NA, Hietanen MA, Ibbotson MR (2006). Neurons in V1, V2, and PMLS of cat cortex are speed tuned but not acceleration tuned: The influence of motion adaptation. Journal of Neurophysiology.

[CR35] Price NSC, Ono S, Mustari MJ, Ibbotson MR (2005). Comparing acceleration and speed tuning in macaque MT: Physiology and modeling. Journal of Neurophysiology.

[CR36] Priebe NJ, Lisberger SG (2002). Constraints on the source of short-term motion adaptation in macaque area MT. Journal of Neurophysiology.

[CR37] Qian N, Andersen RA, Adelson EH (1994). Transparent motion perception as detection of unbalanced motion signals. I. Psychophysics. The Journal of Neuroscience.

[CR38] Raizada RDS, Grossberg S (2001). Context-sensitive binding by the laminar circuits of V1 and V2: A unified model of perceptual grouping, attention, and orientation contrast. Visual Cognition.

[CR39] Ratcliff R (1978). A theory of memory retrieval. Psychological Review.

[CR40] Rubin E, Beardslee DC, Wertheimer M (1921). Visuaell wahrgenommene Figuren [figure and ground]. Readings in perception.

[CR41] Runeson S (1974). Constant velocity — not perceived as such. Psychological Research.

[CR42] Schlack A, Krekelberg B, Albright TD (2007). Recent history of stimulus speeds affects the speed tuning of neurons in area MT. The Journal of Neuroscience.

[CR43] Shadlen MN, Newsome WT (2001). Neural basis of a perceptual decision in the parietal cortex (area LIP) of the rhesus monkey. Journal of Neurophysiology.

[CR44] Shipley TF, Kellman PJ (1994). Spatiotemporal boundary formation: Boundary, form, and motion perception from transformations of surface elements. Journal of Experimental Psychology: General.

[CR45] Simoncelli EP, Heeger DJ (1998). A model of neuronal responses in visual area MT. Vision Research.

[CR46] Srinivasan, M. V., Lehrer, M., & Horridge, G. A. (1990). Visual figure-ground discrimination in the honeybee: The role of motion parallax at boundaries. *Proceedings of the Royal Society of London. Series B, Biological Sciences, 238*(1293), 331–350.

[CR47] Tadin D, Lappin JS, Blake R, Glasser DM (2010). High temporal precision for perceiving event offsets. Vision Research.

[CR48] Thiel A, Greschner M, Eurich CW, Ammermüller J, Kretzberg J (2007). Contribution of individual retinal ganglion cell responses to velocity and acceleration encoding. Journal of Neurophysiology.

[CR49] Thompson P (1982). Perceived rate of movement depends on contrast. Vision Research.

[CR50] van Doorn AJ, Koenderink JJ (1982). Spatial properties of the visual detectability of moving spatial white noise. Experimental Brain Research.

[CR51] van Doorn, A. J., Koenderink, J. J., & van de Grind, W. A. (1984). Limits in spatio-temporal correlation and the perception of visual movement. In A. J. van Doorn, W. A. van de Grind, & J. J. Koenderink (Eds.), *Limits in perception* (pp. 203–234). VSP.

[CR52] Yonas A, Craton LG, Thompson WB (1987). Relative motion: Kinetic information for the order of depth at an edge. Perception & Psychophysics.

[CR53] Zhang C, Wang Y-J, Qi X-L (2005). Modeling the acceleration sensitive neurons in the pigeon optokinetic system. Biological Cybernetics.

